# Correlations between SHBG, Sex Hormones, Inflammation, and Neurocognitive Decline in Alzheimer's Disease: A Retrospective Study

**DOI:** 10.2174/0115672050341904241111082935

**Published:** 2024-11-25

**Authors:** Jiali Jin, Libo Lu, Kaiyao Hua, Ling Fang, Xiao Li, Wen Li

**Affiliations:** 1 *Department of Neurology*, *Shanghai Yangpu District Kongjiang Hospital, Shanghai, China;*; 2 *Department of Surgery, Shanghai Tenth People’s Hospital, Shanghai, China*

**Keywords:** Correlation analysis, serum hormone binding globulin levels, neurocognitive abilities, Alzheimer's disease, inflammation, retrospective

## Abstract

**Background:**

Alzheimer's Disease (AD) is characterized by a progressive neurodegenerative process leading to cognitive decline and functional impairment. Endocrine factors, particularly sex hormones and their binding proteins, play a critical role in AD pathophysiology. Understanding the relationship between these factors and AD is essential for developing targeted interventions.

**Objective:**

To investigate the potential links between sex hormone binding globulin (SHBG) levels, sex hormone profiles, inflammatory markers, and neurocognitive decline in patients with AD.

**Methods:**

A retrospective case-control investigation was conducted with 110 AD patients who were admitted to our hospital from January 2021 to December 2023, and the patients were classified into either a mild neurocognitive impairment group (n=59) or a moderate to severe neurocognitive impairment group (n=51) according to their cognitive function. Correlation and regression analyses were conducted to examine relationships between variable factors.

**Results:**

The study revealed a significant neurocognitive decline in AD patients with lower Mini-Mental State Examination (MMSE) and higher AD Assessment Scale-Cognitive Subscale (ADAS-Cog) scores in the moderate to severe neurocognitive impairment group compared to the mild neurocognitive impairment group. Additionally, the moderate to severe neurocognitive impairment group significantly increased for SHBG, estradiol, progesterone inflammatory markers [C-reactive protein (CRP), interleukin-6 (IL-6), tumor necrosis factor α (TNF-α), interleukin-1β (IL-1β). It decreased for follicle-stimulating hormone (FSH) and luteinizing hormone (LH)]. Moreover, significant positive correlations were found between SHBG levels and ADAS-Cog scores, and significant negative correlations were found between SHBG levels and MMSE scores. FSH showed significant negative correlations with the MMSE score, while certain inflammatory markers demonstrated significant correlations with neurocognitive abilities. The correlation between sex hormones and inflammatory factors is weak. FSH, LH, SHBG, CRP, IL-6, TNF-α, and IL-1β are risk factors for neurocognitive impairment, while E2 and P are protective factors.

**Conclusion:**

The study provides evidence of significant correlations between SHBG levels, sex hormone profiles, inflammatory markers, and neurocognitive decline in AD patients.

## 1 INTRODUCTION

Alzheimer's Disease (AD) is a significant and increasing public health concern, defined by progressive neurocognitive decline, memory impairment, and functional disability [1-3]. According to the World Health Organization (WHO), around 50 million people globally have dementia, with 60-70% of cases linked to AD [4, 5]. With the global aging trend, these numbers were expected to escalate, posing substantial societal and healthcare challenges. AD significantly impacts older adults, with prevalence doubling every five years after age 65, thereby increasing the burden of disability in the elderly population [6]. Underscoring the urgency of understanding the intricate pathophysiological mechanisms underlying cognitive deterioration in this devastating neurodegenerative disorder. Although the neuropathological hallmarks of AD, such as beta-amyloid plaques and neurofibrillary tangles, have been widely investigated [7-9], recent research has drawn attention to the potential impact of sex hormones and inflammatory processes on neurocognitive function in AD.

The intricate relationships between sex hormone binding globulin (SHBG) levels, sex hormones, inflammatory markers, and their potential impact on neurocognitive decline in AD remain an area of active investigation [10, 11]. SHBG, a glycoprotein that binds to sex hormones, such as testosterone and estradiol, plays a crucial role in regulating the bioavailability and activity of these hormones [12, 13]. Recent studies have suggested that dysregulation in sex hormone levels, potentially mediated by altered SHBG levels, may contribute to synaptic dysfunction, neuroinflammation, and, ultimately, cognitive impairment in AD [14]. Specifically, SHBG affects the bioavailability of sex hormones by binding to them and altering their free concentrations in the bloodstream. This regulation is critical because sex hormones, particularly estradiol, have been shown to exert neuroprotective effects by influencing synaptic plasticity, neurogenesis, and neuronal survival [15]. Reduced levels of SHBG can lead to lower bioavailability of these neuroprotective hormones, compromising their beneficial effects and potentially contributing to neurodegeneration and cognitive decline in AD [16]. Additionally, persistent neuroinflammation, marked by the release of pro-inflammatory cytokines and activation of glial cells, has been linked to worsening synaptic dysfunction, neuronal injury, and cognitive decline in AD [17-19]. However, the complex interplay between sex hormones, SHBG, inflammatory markers, and neurocognitive abilities in the context of AD pathogenesis remains incompletely understood.

Previous studies have shown that alterations in sex hormone levels and inflammatory markers may contribute to synaptic dysfunction and cognitive impairment in AD [20-22]. Despite advances in understanding these mechanisms, the specific roles of SHBG and sex hormones in AD remain poorly understood. This exploratory study aims to investigate the potential associations between SHBG levels, sex hormone profiles, inflammatory markers, and neurocognitive decline in AD patients. By elucidating these relationships, we hope to provide new insights into the underlying mechanisms of AD and identify potential therapeutic targets.

## 2 MATERIALS AND METHODS

### 2.1 Study Design

In this retrospective case-control investigation, 110 individuals diagnosed with AD were selected from patients admitted to our hospital from January 2021 to December 2023. They were then classified into either a mild neurocognitive impairment group (n=59) or a moderate to severe neurocognitive impairment group (n=51) according to their cognitive function.

This study was approved by the Ethics Committee of Shanghai Tenth People’s Hospital in accordance with regulatory and ethical guidelines pertaining to retrospective research studies (No. LL-2020-KY-24). Informed consent was waived for this retrospective study due to the exclusive use of de-identified patient data, which posed no potential harm or impact on patient care.

### 2.2 Inclusion and Exclusion Criteria

#### 2.2.1 Inclusion Criteria

(1) Diagnosis of AD based on clinical criteria, including comprehensive cognitive assessments, neuroimaging (such as Magnetic Resonance Imaging (MRI) and Positron Emission Tomography (PET) scans to identify amyloid plaques and neurofibrillary tangles) [23]; (2) Male sex; (3) Non-pregnant, non-lactating, postmenopausal, or surgically sterilized female; (4) Age of 60 years or above; (5) Possession of complete medical records.

#### 2.2.2 Exclusion Criteria

(1) Significant neurological disorders apart from AD (*e.g.,* hypoxia, stroke, or traumatic brain injury); (2) an active and uncontrolled seizure disorder, severe unstable cardiovascular disease, a disability impairing their ability to meet study requirements (*e.g.,* blindness, deafness, severe language difficulty); (3)a history of head injury with loss of consciousness exceeding 1 hour; (4) a history of prostate cancer, myocardial infarction, abnormal renal or hepatic disease, prior testosterone or other sex hormone therapy, or other gonadal endocrine disorders; (5) Diagnosed with vascular dementia, dementia with Lewy bodies, normal pressure hydrocephalus and other forms of dementia, chronic inflammatory diseases, and the use of immunomodulatory drugs or medications known to affect the immune profile.

### 2.3 Grouping Criteria

The classification process involved the use of the Mini-Mental State Examination (MMSE) and AD Assessment Scale-Cognitive Subscale (ADAS-Cog) tools. Patients' scores on these two scales were analyzed, with scores ranging from 21 to 26 on the MMSE scale and ≤22 on the ADAS-Cog scale defining the mild neurocognitive impairment group, and scores of ≤20 on the MMSE scale and >22 on the ADAS-Cog scale defining the moderate to severe neurocognitive impairment group.

The MMSE was a commonly used neuropsychological test for evaluating a patient's intellectual status and cognitive impairment. The assessment results in a scoring system out of 30 points. Scores between 27 and 30 suggest normal cognitive function without any impairment. Mild cognitive impairment is linked to scores ranging from 21 to 26, while moderate cognitive impairment corresponds to scores between 10 and 20. Scores of 9 points or fewer indicate severe cognitive impairment. Cronbach alpha coefficient for MMSE was 0.71 [24].

The ADAS-Cog scale was used as an assessment tool to assess cognitive function in patients with mild cognitive impairment and AD. It consists of 12 items divided into cognitive and non-cognitive sections. Professionals typically conducted the scoring and usually takes about 45 minutes. Scores range from 0 (no errors or impairments) to a maximum of 70 (severe impairment across all test items). In mild AD patients, the threshold for the ADAS-Cog was 22. Higher scores indicate more severe cognitive decline in patients. The reliability of the ADAS-Cog scale was reported to be 0.843 [25].

### 2.4 Data Collection

The patients' general data, including education level, sex, body mass index (BMI), age, smoking history, hypertension, alcohol consumption history, diabetes, hyperlipidemia, family history of AD, and illness duration, were gathered from the medical records system. Illness duration was estimated based on the earliest documented report of cognitive symptoms and confirmed through caregiver reports. The serum hormone levels of the two groups of patients were measured and analyzed, including estradiol, follicle-stimulating hormone (FSH), progesterone, luteinizing hormone (LH), testosterone, and SHBG levels. Additionally, the levels of inflammatory markers in patients were measured and recorded, which included C-reactive protein (CRP), interleukin-6 (IL-6), tumor necrosis factor α (TNF-α), interleukin-1β (IL-1β), and interleukin-10 (IL-10).

### 2.5 Serum Hormone Levels

Patients provided fasting blood samples (5ml) between 8-9 am, which were then centrifuged at 3000r/min for 10 minutes using a high-speed, low-temperature centrifuge. The serum was isolated and kept at -30°C for analysis. Following this, the levels of estradiol, testosterone, progesterone, FSH, LH, and SHBG were measured. Testosterone and estradiol assays were performed using a double-antibody method provided by Tianjin Depot Company and conducted following the manufacturer's instructions. The testing instrument used was the SN-682 radioimmunoassay counter produced by Shanghai Nuclear-Fox Optoelectronics Instrument Co., Ltd. Serum progesterone, FSH, and LH levels were determined using the IMMULITE automated chemiluminescent immunoassay system and associated reagents from DPC, USA. Serum SHBG levels were measured using an electrochemiluminescence assay (Siemens, Germany).

### 2.6 Inflammatory Markers Levels

Fasting venous blood samples (5mL) were collected from the patients in the morning and allowed to stand at room temperature for 30 minutes. Following this, the samples underwent centrifugation at 3000r/min for 10 minutes using a high-speed, low-temperature centrifuge. The resulting serum's upper layer was then stored at -80°C for subsequent testing. The quantification of CRP, IL-6, TNF-α, IL-1β, and IL-10 levels in the patients was performed using enzyme-linked immunosorbent assay (ELISA).

For the CRP assay, the Siemens BN II or BN Pro specific protein analyzer (Siemens, Germany) and original matching reagents (batch number 16573C) were used based on the principle of immunonephelometry. The IL-6 assay kit was obtained from BioSino Bio-Technology & Science Inc., the TNF-α assay kit was obtained from BioSino Bio-Technology & Science Inc., the IL-1β assay kit was obtained from Beijing Elabscience Biotechnology Co., Ltd.. The testing instrument used was the RT-2100C automatic enzyme immunoassay analyzer (Shenzhen Rayto Life and Analytical Sciences Co., Ltd.). The IL-10 assay was obtained from ADL, USA, and the enzyme immunoassay analyzer was obtained from MK-3, Finland. Specific operations were conducted according to the instructions.

### 2.7 Sample Size

The parameter estimates for the sample size are based on a previous large-scale clinical trial targeting Alzheimer's disease [ 26 ]. We performed sample size calculations using G*Power 3.1.9.7 software, with a two-sided significance threshold (α) of 0.05 and power set at 0.80, resulting in a required sample size of 45 per group. Assuming a 10% loss to follow-up, we conservatively estimate that at least 50 patients per group are needed.

### 2.8 Statistical Methods

The data was analyzed using SPSS 29.0 statistical software (SPSS Inc, Chicago, IL, USA). Categorical variables were represented as [n (%)] and subjected to chi-square testing. The normality of continuous variables was assessed with the Shapiro-Wilk test. Variables displaying a normal distribution were expressed as (Mean±SD) and evaluated using the corrected variance t-test. Conversely, non-normally distributed continuous variables were depicted as median with the interquartile range (25^th^ percentile, 75^th^ percentile) and analyzed utilizing the Wilcoxon rank-sum test. Statistical significance was established at *P*<0.05 for a two-tailed test. For continuous variables, Pearson correlation analysis was used to examine their relationships, while Spearman correlation analysis was used to assess the relationships between categorical variables and other indicators.

## 3 RESULTS

### 3.1 Demographic Characteristics

In this study, the MMSE score for the mild neurocognitive impairment group is 23.45 ± 0.87, and the ADAS-Cog score is 15.21 ± 3.45. The MMSE score for the moderate to severe neurocognitive impairment group is 15.32 ± 1.49, and the ADAS-Cog score is 32.89 ± 5.67. The mild and moderate to severe neurocognitive impairment groups were compared through independent sample t-tests and chi-square tests. No significant differences were found in age, sex, BMI, education level, smoking history, alcohol history, hypertension prevalence, diabetes prevalence, hyperlipidemia prevalence, and family history of AD between the two groups (*P*>0.05) (Table **1**). However, the disease duration in the moderate to severe neurocognitive impairment group was significantly longer than that in the mild neurocognitive impairment group(5.67±1.23 *vs.* 2.56±0.98, t=14.749, *P*<0.001).

### 3.2 Serum Sex Hormone Levels

In examining serum sex hormone levels, the moderate to severe neurocognitive impairment group showed statistically significant differences in SHBG levels compared to the mild neurocognitive impairment group (28.75 ± 3.52 nmol/L *vs.* 24.51 ± 3.17 nmol/L, t=6.598, *P*<0.001) (Table **2**). Also, the moderate to severe neurocognitive impairment group exhibited statistically significant differences in estradiol (38.67 ± 14.35 pg/mL *vs.* 32.14 ± 15.21 pg/mL, t=2.305, *P*=0.023), progesterone (21.65 ± 7.78 pg/mL *vs.* 17.89 ± 8.76 pg / mL, t =2.368, *P* =0.020), follicle-stimulating hormone (8.84 ± 2.34 pg/mL *vs.* 10.02 ± 3.21 pg / mL, t =2.164, *P* =0.033), and LH levels (6.94 ± 2.38 pg / mL *vs.* 7.89 ± 2.56 pg / mL, t =2.005, *P*=0.048) when compared to the mild neurocognitive impairment group. Conversely, no statistically prominent differences were observed in testosterone (511.87 ± 45.32 pg/mL *vs.* 497.21 ± 35.67 pg/mL, t=1.896, *P*=0.061) between the two groups. These findings highlight the potential association between serum sex hormone levels and the decline in neurocognitive abilities in patients with AD.

### 3.3 Inflammatory Marker Levels

In assessing the inflammatory marker levels, the moderate to severe neurocognitive impairment group exhibited statistically significant differences in CRP (3.67 ± 1.52 mg/L *vs.* 2.95 ± 1.73 mg/L, t=2.329, *P*=0.022), IL-6 (5.58 ± 1.89 pg/mL *vs.* 4.76 ± 2.05 pg/mL, t=2.179, *P*=0.031), TNF-α (15.67 ± 4.86 pg/mL *vs.* 13.73 ± 3.54 pg/mL, t=2.362, *P*=0.020), and interleukin-1β levels (3.45 ± 1.12 pg/mL *vs.* 2.95 ± 1.27 pg/mL, t=2.215, *P*=0.029) when compared to the mild neurocognitive impairment group (Table **3**). However, no statistically prominent variations in interleukin-10 levels were detected between the two groups. (4.32 ± 1.67 pg/mL *vs.* 3.88 ± 1.01 pg/mL, t=1.680, *P*=0.096). These findings suggested a potential correlation between elevated levels of certain inflammatory markers and the decline in neurocognitive abilities in AD patients.

### 3.4 Correlation Analysis

**Fig. (1):**
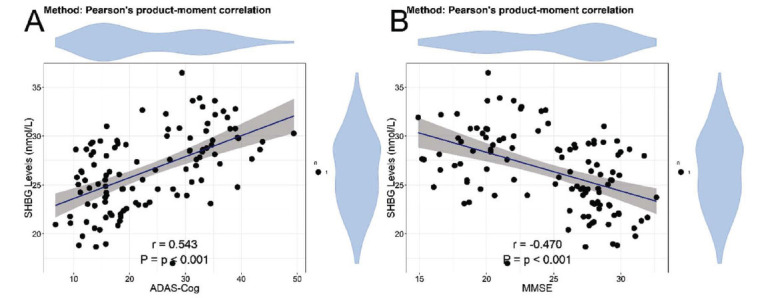
Correlation between neurocognitive abilities and SHBG levels. (**A**) Correlation between ADAS-Cog and SHBG levels. (**B**) Correlation between MMSE and SHBG levels. **Abbreviations**: SHBG: serum hormone binding globulin; MMSE: Mini-Mental State Examination; ADAS-Cog: AD Assessment Scale-Cognitive Subscale.

**Fig. (2):**
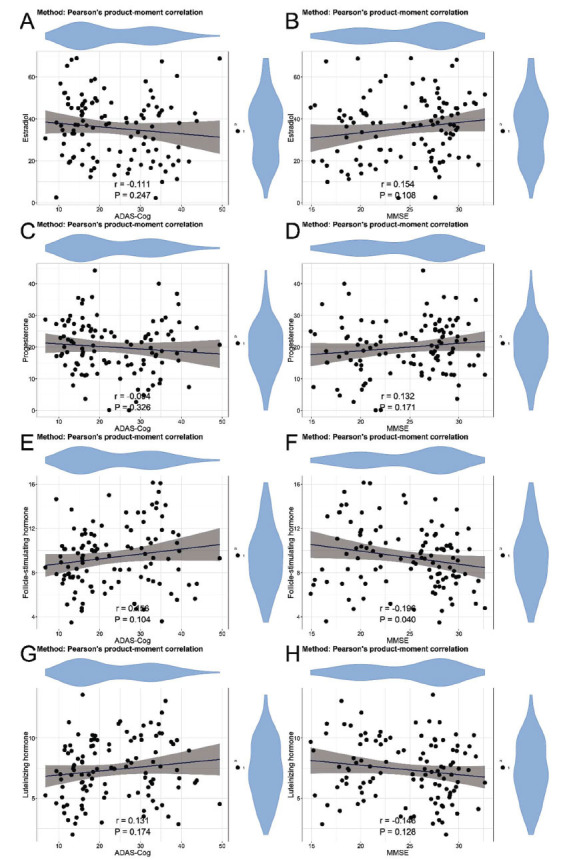
Correlation between neurocognitive abilities and serum sex hormone levels. (**A**) Correlation between ADAS-Cog and estradiol. (**B**) Correlation between MMSE and estradiol. (**C**) Correlation between ADAS-Cog and progesterone. (**D**) Correlation between MMSE and progesterone. (**E**) Correlation between ADAS-Cog and FSH. (**F**) Correlation between MMSE and FSH. (**G**) Correlation between ADAS-Cog and LH. (**H**) Correlation between MMSE and LH. **Abbreviations**: MMSE: Mini-Mental State Examination; ADAS-Cog: AD Assessment Scale-Cognitive Subscale; FSH: follicle-stimulating hormone; LH: luteinizing hormone.

**Fig. (3):**
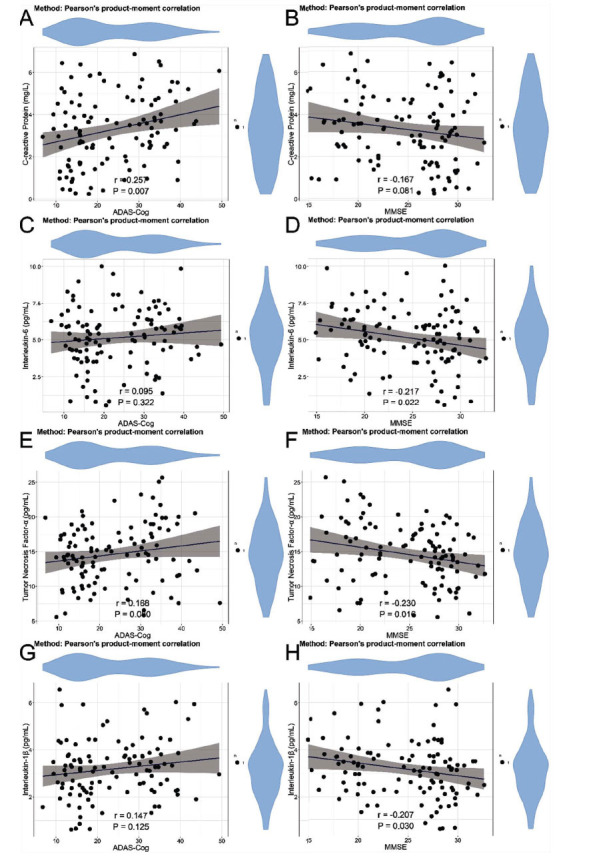
Correlation between neurocognitive abilities and inflammatory marker levels. (**A**) Correction between ADAS-Cog and CRP. (**B**) Correction between MMSE and CRP. (**C**) Correction between ADAS-Cog and IL-6. (**D**) Correction between MMSE and IL-6. (**E**) Correction between ADAS-Cog and TNF-α. (**F**) Correction between MMSE and TNF-α. (**G**) Correction between ADAS-Cog and IL-1β. (**H**) Correction between MMSE and IL-1β. **Abbreviations**: MMSE: Mini-Mental State Examination; ADAS-Cog: AD Assessment Scale-Cognitive Subscale; CRP: C-reactive protein; IL-6: interleukin-6; TNF-α: tumor necrosis factor α; IL-1β: interleukin-1β.

In the correlation analysis of neurocognitive abilities and indicators in patients with AD, significant positive correlations were found between SHBG and ADAS-Cog (r = 0.543, *P* < 0.001) (Fig. **1A**), and significant negative correlations were found between SHBG and MMSE (r = -0.470, *P* < 0.001) (Fig. **1B**), indicating an association between SHBG levels and cognitive abilities. No statistically significant correlations were found between ADAS-Cog and estradiol (r = -0.111, *P* = 0.247) (Fig. **2A**), MMSE and estradiol (r = 0.154, *P* = 0.108) (Fig. **2B**), ADAS-Cog and progesterone (r = -0.094, *P* = 0.326) (Fig. **2C**), MMSE and progesterone (r = 0.132, *P* = 0.171) (Fig. **2D**), ADAS-Cog and FSH (r = 0.156, *P* = 0.104) (Fig. **2E**). Significant negative correlation was found between MMSE and FSH (r = -0.196, *P* = 0.040) (Fig. **2F**). No statistically significant correlations were found between ADAS-Cog and LH (r = 0.131, *P* = 0.174) (Fig. **2G**), MMSE and LH (r = -0.164, *P* = 0.128) (Fig. **2H**). Moreover, significant positive correlation was found between ADAS-Cog and CRP (r = 0.257, *P* = 0.007) (Fig. **3A**). No statistically significant correlations were found between MMSE and CRP (r = -0.167, *P* = 0.081) (Fig. **3B**), ADAS-Cog and IL-6 (r = 0.095, *P* = 0.322) (Fig. **3C**). Significant negative correlation was found between MMSE and IL-6 (r = -0.217, *P* = 0.022) (Fig. **3D**). No statistically significant correlation was found between ADAS-Cog and TNF-α (r = 0.168, *P* = 0.080) (Fig. **3E**). Significant negative correlation was found between MMSE and TNF-α (r = -0.230, *P* = 0.016) (Fig. **3F**). No statistically significant correlation was found between ADAS-Cog and IL-1β (r = 0.147, P=0.125) (Fig. **3G**). Significant negative correlation was found between MMSE and IL-1β (r=-0.207, P=0.030) (Fig. **3H**). In addition, we analyzed the correlation between sex hormones and inflammatory factors. The results showed that the correlations between various sex hormones and inflammatory factors were weak (rho values all < 0.3) (Table **4**), indicating a low level of association between them.

### 3.5 Logistic Regression Analysis

Logistic regression analysis showed that the OR values for FSH, LH, SHBG, CRP, IL-6, TNF-α, and IL-1β were > 1, indicating they are risk factors for neurocognitive impairment, while the OR values for E2 and P were < 1, suggesting they are protective factors (Table **5**). This indicates a significant association between sex hormones and inflammatory factors with the severity of neurocognitive impairment.

## 4 DISCUSSION

AD is a neurodegenerative condition marked by a progressive decline in cognition, memory, and the capability to perform daily tasks [27, 28]. The complex interplay between neurobiological, hormonal, and inflammatory mechanisms in AD pathophysiology has been the subject of extensive investigation [29, 30]. Our study revealed significant differences in SHBG levels, sex hormone profiles, and inflammatory markers between mild and moderate to severe neurocognitive impairment groups in AD patients. Specifically, patients with moderate to severe neurocognitive impairment showed significantly higher SHBG, estradiol, progesterone, CRP, IL-6, TNF-α, and IL-1β levels and significantly lower FSH and LH levels compared to those with mild neurocognitive impairment.

The characteristics of the patients were comparable between the mild and moderate to severe neurocognitive impairment groups. Our results demonstrated that the moderate to severe group exhibited significantly lower scores on the MMSE and observably higher scores on the ADAS-Cog in comparison to the mild group. The disease duration in the moderate to severe group was significantly longer than that in the mild group. These findings confirm the presence of neurocognitive decline in patients with AD, consistent with the progressive nature of the disease and the expected trajectory of cognitive deterioration over time.

A notable correlation was noted between SHBG levels and both MMSE and ADAS-Cog scores, suggesting that SHBG levels were linked to neurocognitive changes. Significant positive correlations were found between SHBG levels and ADAS-Cog scores, and significant negative correlations were found between SHBG levels and MMSE scores. SHBG, as a glycoprotein, has the function of binding to sex hormones like testosterone and estradiol, thereby playing a role in regulating their bioavailability and activity. This observation suggests a potential link between sex hormone dysregulation and cognitive impairment in AD, supporting previous research indicating the involvement of sex hormones in neuroprotection and cognitive function [31, 32]. The impaired adjustment of sex hormone levels may impact cellular resilience, neurotransmitter function, and neuronal plasticity, thereby influencing neurocognitive decline in AD [33-35].

The study also revealed significant associations between certain sex hormone levels and neurocognitive abilities. Specifically, FSH showed significant negative correlations with MMSE, highlighting the potential influence of hormones on cognitive function in AD. Previous studies [36, 37] have reported the neuroprotective effects of FSH, emphasizing their role in synaptic plasticity, neurogenesis, and neuronal survival. Our findings support the notion that sex hormone imbalances play a role in the neurocognitive decline seen in AD, potentially opening avenues for hormone-based interventions in AD management. Persistent neuroinflammation is a notable characteristic of AD, marked by the activation of microglia and astrocytes, along with the release of pro-inflammatory cytokines [38].

Inflammatory markers, including CRP, interleukins, and tumor necrosis factor, were associated with neuroinflammatory processes, which can exacerbate synaptic dysfunction, promote neuronal injury, and contribute to cognitive decline [39, 40]. The correlations observed between inflammatory markers and neurocognitive abilities suggest that heightened neuroinflammatory responses may play a role in the progression of cognitive impairment in AD. Our analysis identified noteworthy associations between inflammatory markers and neurocognitive decline in AD patients. CRP exhibited significant positive correlations with ADAS-Cog. IL-6, TNF-α, and interleukin-1β showed significant negative correlations with MMSE. These findings suggested a potential role for inflammation in exacerbating cognitive impairment. Persistent neuroinflammation was a recognized characteristic of AD pathogenesis, contributing to synaptic dysfunction, neurodegeneration, and cognitive decline [41, 42]. The observed associations between inflammatory markers and neurocognitive abilities underscore the complex interplay between inflammatory processes and cognitive function in AD.

The results of this study offer valuable insights into the multifaceted mechanisms that underlie neurocognitive decline in AD. The correlation analysis revealed intricate relationships between sex hormone-binding globulin levels, sex hormones, inflammatory markers, and neurocognitive abilities. However, correlation analysis of sex hormones and inflammatory factors showed that their correlations were low, indicating that they may act relatively independently in the process of AD, affecting neural cognitive function. Current evaluations of the efficacy of hormonal and anti-inflammatory treatments in AD patients suggest promising avenues for therapeutic intervention. For instance, hormone replacement therapies (HRT) involving estrogen has shown potential benefits in improving cognitive function and reducing the risk of AD in postmenopausal women [43-45]. Similarly, anti-inflammatory agents, such as nonsteroidal anti-inflammatory drugs (NSAIDs) and corticosteroids, have been explored for their potential to reduce neuroinflammation and slow cognitive decline [46, 47]. These findings highlight the potential of these treatment strategies and warrant further investigation to confirm their efficacy and safety profiles.

Notably, recent studies have emphasized the importance of the blood-brain barrier (BBB) in AD [48]. The integrity of the BBB plays a crucial role in maintaining the homeostasis of the central nervous system and protecting it from systemic influences [49, 50]. Specifically, alterations in BBB integrity can allow systemic markers, such as sex hormones and inflammatory molecules, to enter the brain parenchyma, potentially contributing to neuroinflammation and cognitive decline [51, 52]. Our findings suggested that SHBG levels, sex hormones, and inflammatory markers are associated with neurocognitive decline in AD. Given the role of BBB in regulating the entry of these systemic markers into the brain, it is plausible that changes in BBB permeability could modulate the impact of these biomarkers on neurocognitive function [53, 54]. Future research should explore the interactions between BBB integrity and the biomarkers studied here to gain a more comprehensive understanding of their roles in AD pathophysiology.

Despite the significant correlations observed between SHBG levels, sex hormones, and inflammatory markers with neurocognitive decline, some variables, such as testosterone and certain inflammatory markers (*e.g.,* IL-10), did not show significant correlations. This lack of significant findings could be attributed to several factors. Firstly, the sample size of the study may not have been sufficiently large to detect subtle differences in hormone levels and inflammatory markers. Secondly, the natural variability in hormone levels among individuals and the presence of other confounding factors, such as comorbid conditions or medication use, may have influenced the results. Future studies with larger sample sizes and more controlled designs will be necessary to elucidate the true relationships between these variables and neurocognitive decline in AD.

Moreover, several limitations also warrant consideration when interpreting the results of this study. The retrospective design has inherent limitations, including potential selection bias, limited control over data collection, and the inability to establish causal relationships. Furthermore, the cross-sectional nature of the study prevents the assessment of temporal relationships and longitudinal changes in hormone and inflammatory marker levels relative to cognitive decline. Subsequent research efforts should strive to overcome these limitations and provide a deeper understanding of the mechanisms behind the observed correlations. Longitudinal studies were warranted to investigate the temporal dynamics of sex hormone and inflammatory marker levels in relation to neurocognitive decline in AD. This approach would provide valuable insights into the trajectories of hormonal dysregulation and neuroinflammation throughout different stages of AD progression. Furthermore, interventional studies targeting sex hormone modulation and inflammation control were needed to evaluate the potential therapeutic efficacy of hormone-based and anti-inflammatory interventions in mitigating neurocognitive decline in AD. Finally, future studies can expand the sample size, given the effects produced by sex hormones, and perform stratified analyses based on gender to investigate the impact of gender on Alzheimer's disease and neurocognitive impairment.

## CONCLUSION

In conclusion, our study provides evidence of significant correlations between SHBG levels, sex hormone profiles, inflammatory markers, and neurocognitive decline in AD patients. These findings add to the expanding literature that clarifies the complex interaction between hormonal, inflammatory, and neurocognitive factors in AD pathophysiology. The noted links emphasize the potential significance of sex hormones and inflammation in the realm of cognitive impairment in AD, paving the way for additional investigations and potential intervention strategies targeting these interconnected pathways. Ultimately, a deeper understanding of the roles of sex hormones and inflammation in AD may inform novel therapeutic approaches development targeting these mechanisms to potentially mitigate neurocognitive decline and improve clinical outcomes for individuals affected by AD.

## AUTHORS’ CONTRIBUTIONS

The authors confirm their contribution to the paper as follows: study conception and design: JJ, LL; Data Collection: KH; Data Analysis or Interpretation: LF, XL; Writing the paper: WL. All authors reviewed the results and approved the final version of the manuscript.

## Figures and Tables

**Table 1:** Comparison of demographic characteristics.

**Demographic Characteristic**	** Mild Neurocognitive Impairment Group (n=59) **	** Moderate to Severe Neurocognitive Impairment Group (n=51) **	**t/χ^2^**	***P value***
Age (years)	75.21 ± 3.45	76.34 ± 4.21	1.517	0.133
Sex (M/F)	26 (44.07%) / 33 (55.93%)	20 (39.22%) / 31 (60.78%)	0.103	0.748
BMI (kg/m^2^)	26.18 ± 3.01	26.85 ± 2.89	1.193	0.236
Education situation	-	-	0.049	0.825
- Junior high school and below	37 (62.71%)	30 (58.82%)	-	-
- Junior high school or above	22 (37.29%)	21 (41.18%)	-	-
Smoking history (%)	19 (32.2%)	18 (35.29%)	0.020	0.889
Alcohol history	23 (38.98%)	18 (35.29%)	0.041	0.840
Hypertension (%)	17 (28.81%)	11 (21.57%)	0.423	0.515
Diabetes (%)	15 (25.42%)	12 (23.53%)	0	0.994
Hyperlipidemia [n(%)]	14 (23.73%)	11 (21.57%)	0.002	0.967
Family history of AD	14 (23.73%)	10 (19.61%)	0.084	0.772
Disease course (year)	2.56 ± 0.98	5.67 ± 1.23	14.749	<0.001
MMSE	23.45 ± 0.87	15.32 ± 1.49	35.505	< 0.001
ADAS-Cog	15.21 ± 3.45	32.89 ± 5.67	19.385	< 0.001

**Abbreviations:** BMI: body mass index; MMSE: Mini-Mental State Examination; ADAS-Cog: AD Assessment Scale-Cognitive Subscale.>

**Table 2:** Comparison of serum sex hormone levels.

**Hormone Level (pg/mL)**	**Mild Neurocognitive Impairment Group (n=59)**	**Moderate to Severe Neurocognitive Impairment Group (n=51)**	**t**	***P value***
Estradiol(pg/ml)	38.67 ± 14.35	32.14 ± 15.21	2.305	0.023
Testosterone(pg/ml)	511.87 ± 45.32	497.21 ± 35.67	1.896	0.061
Progesterone(pg/ml)	21.65 ± 7.78	17.89 ± 8.76	2.368	0.020
Follicle-stimulating hormone (pg/ml)	8.84 ± 2.34	10.02 ± 3.21	2.164	0.033
Luteinizing hormone (pg/ml)	6.94 ± 2.38	7.89 ± 2.56	2.005	0.048
SHBG Levels (nmol/L)	24.51 ± 3.17	28.75 ± 3.52	6.598	< 0.001

**Abbreviations:** SHBG: serum hormone binding globulin.

**Table 3:** Comparison of inflammatory marker levels.

**Inflammatory Marker**	**Mild Neurocognitive Impairment Group (n=59)**	**Moderate to Severe Neurocognitive impairment Group (n=51)**	**t/χ^2^**	***P value***
C-reactive Protein (mg/L)	2.95 ± 1.73	3.67 ± 1.52	2.329	0.022
Interleukin-6 (pg/mL)	4.76 ± 2.05	5.58 ± 1.89	2.179	0.031
TNF-α(pg/mL)	13.73 ± 3.54	15.67 ± 4.86	2.362	0.020
Interleukin-1β (pg/mL)	2.95 ± 1.27	3.45 ± 1.12	2.215	0.029
Interleukin-10 (pg/mL)	4.32 ± 1.67	3.88 ± 1.01	1.680	0.096

**Table 4:** Correlation between sex hormone and inflammatory marker.

**Variable**	**E2**	**P**	**FSH**	**LH**	**SHBG**	**CRP**	**IL-6**	**TNF-α**	**IL-1β**
**E2**		0.116	-0.122	-0.128	0.074	0.071	-0.117	-0.023	0.024
**P**			-0.049	0.112	-0.037	-0.092	-0.029	0.083	-0.289
**FSH**				0.054	0.117	-0.017	0.078	0.197	-0.062
**LH**					0.027	0.043	-0.078	0.223	0.047
**SHBG**						0.204	0.068	-0.036	0.095
**CRP**							0.010	0.099	-0.097
**IL-6**								0.150	0.114
**TNF-α**									-0.038
**IL-1β**									

**Note:** E2: estradiol; P: progesterone; FSH: follicle-stimulating hormone; LH: luteinizing hormone; SHBG: sex hormone-binding globulin; CRP: C-reactive protein; IL-6: interleukin-6; TNF-α: tumor necrosis factor α; IL-1β: interleukin-1β.

**Table 5:** Logistic regression analysis of various indicators with the severity of neurocognitive impairment.

**Parameter**	**SE**	**Wald**	**OR**	**95% CI**	***P* value**
E2	0.024	-2.775	0.936	0.893-0.981	0.006
P	0.038	-1.604	0.941	0.873-1.014	0.039
FSH	0.114	1.469	1.183	0.945-1.479	0.042
LH	0.127	1.018	1.138	0.888-1.458	0.039
SHBG	0.126	4.568	1.774	1.387-2.269	<0.001
CRP	0.190	1.682	1.377	0.949-2.000	0.042
IL-6	0.156	1.234	1.212	0.893-1.644	0.037
TNF-α	0.084	2.679	1.251	1.062-1.473	0.007
IL-1β	0.254	1.578	1.492	0.908-2.452	0.035

**Note:** E2: estradiol; P: progesterone; FSH: follicle-stimulating hormone; LH: luteinizing hormone; SHBG: sex hormone-binding globulin; CRP: C-reactive protein; IL-6: interleukin-6; TNF-α: tumor necrosis factor α; IL-1β: interleukin-1β; SE: Standard Error; OR: Odds Ratio; CI: Confidence Interval.

## Data Availability

All data generated or analyzed during this study are included in this published article.

## LIST OF ABBREVIATIONS

AD: Alzheimer's Disease
MMSE: Mini-mental State Examination
FSH: Follicle-stimulating Hormone
LH: Luteinizing Hormone
WHO: World Health Organization
SHBG: Sex Hormone Binding Globulin
MRI: Magnetic Resonance Imaging
PET: Positron Emission Tomography
BMI: Body Mass Index
HRT: Hormone Replacement Therapies
NSAIDs: Nonsteroidal Anti-inflammatory Drugs
BBB: Blood-brain Barrier

## STANDARDS OF REPORTING

STROBE guidelines were followed.
